# Proposing a nasal trehalose‐induced autophagy approach against SARS‐CoV 2

**DOI:** 10.1002/hsr2.375

**Published:** 2021-09-27

**Authors:** Mojde Pajokh, Mohammad Pourfridoni

**Affiliations:** ^1^ Department of Anatomical Sciences, School of Medicine Jiroft University of Medical Sciences Jiroft Iran; ^2^ Student Research Committee Jiroft University of Medical Sciences Jiroft Iran

Severe acute respiratory syndrome coronavirus 2 (SARS‐CoV‐2) new variants potently create a further pandemic of coronavirus disease 2019 (COVID‐19), which currently causes global terror.^1^ Beyond an ideal COVID‐19 vaccine with long‐term humoral and cellular memory, mucosal immunity is the right target to protect from complications of a new virus outbreak.^2^

Respiratory damage is the most fatal complication of COVID‐19 patients; the coronaviruses invade nasal epithelium through the high co‐expression levels of angiotensin‐converting‐enzyme 2 (ACE2) and transmembrane protease serine 2 (TMPRSS2), as transmembrane SARS‐CoV 2 receptors, it uses nasal epithelium not only for penetration but also as the main target.^3^ The nasal cavity and neighboring nasopharynx‐associated lymphoid tissue (NALT), as mucosal immunity compartments, has critical protection and homeostasis roles against airborne pathogens.^4^ The specialized olfactory neuroepithelium with SARS‐CoV 2 receptor expression and a high level of direct communication through the axons of the olfactory receptor neurons that pass cribriform foramina in the roof of the nasal cavity and directly reaches the olfactory bulb as the neuronal structure of the brain is therapeutically valuable.^5^ Interestingly, the efferent olfactory axons from the olfactory bulb arrive directly to the brain, and there are abundant centrifugal axons that give rise from different parts of the brain to the olfactory bulb.^6^ In addition to respiratory symptoms, the neurological manifestations of COVID‐19 patients also are prevalent, and anosmia is one of the initial presentations of coronavirus infection; unfortunately, it leads to permanent defect by olfactory bulb atrophy.^7^

Autophagy as recycling lysosome dependent subcellular process is vital for the virus clearance, initiation of innate immune responses, and coordination of adaptive immunity in the host, hence some of the latest viruses evolution is able to serve autophagy machinery for their replication, autophagic flux starts with producing double‐membrane structure, phagophore, expanded to form autophagosome, the targeted cytoplasmic substrate then engulfs by autophagosome and after final autophagosomal maturation join lysosome to degrade its contents, remarkably coronaviruses induces a key regulator of autophagy, autophagy‐related gene 5, but prevent autophagosome maturation at lysosome fusion stage.^8^ The autophagy induction by fasting in the host to upgrade cellular and humoral immunity is proposed as an alternative COVID‐19 preventive approach, hence there is a need to determine the duration of the fasting program and its effect on autophagy.^9^ Trehalose as a safe autophagy inducer agent must be a good candidate for autophagy inducing the natural source disaccharide, which is composed of two glucose α‐1,1‐glycosidic bonds and is a food and drug administration approved product.^10^ Since trehalose effectively induces autophagy and is capable of direct action on the lysosome, the therapeutic value of it must be regarded against viruses that disrupt the autophagy process in the lysosome fusion stage and recently suggested to prevention and restriction of transmission in the SARS‐CoV 2 pandemic.^11^ Several mechanisms have been proposed on how trehalose acts against SARS‐CoV‐2; first, it blocks viruses' entrance to the host cell by inhibition of cathepsin activation during endosomal membrane, and the second inhibit virus replication and maturation by induction of interferon response genes in the host cells.^12^, ^13^ Also, trehalose, as a novel therapeutic strategy in occupational lung disease, reduces apoptosis and inflammatory cytokines through induction of autophagy.^14^ According to Garmise et al, lyophilized trehalose as a mucoadhesive compound is applied in nasal influenza vaccine designing,^15^ and also trehalose was used previously as oral consumption and eye drop,^16^, ^17^ there is no toxicity for mucosal use of trehalose in humans; thus, intranasal mucosal delivery of it must be regarded for prevention, treatment of mild to moderate form of the disease, and long‐term use to remedy permanent defects during and after pandemic situations.

In conclusion, we propose a nasal trehalose delivery method against respiratory and neurological COVID‐19 complications with potential preventive and therapeutic properties during and after infection based on induction of disturbed autophagy cell renewal mechanism that plays critical regulatory roles in mucosal immunity and neural homeostasis. Also, because of the absence of an exact COVID‐19 treatment, complementary and alternative therapeutic approaches that affect various aspects of the disease are urgent in the current position. Some of these approaches are easily accessible, such as a healthy diet, getting enough vitamin C, and consumption of some herbs.^18^, ^19^

Despite studies that suggest the use of trehalose, there is still insufficient information on its safety and effectiveness in children, adults, and the elderly. Also, an animal study shows exacerbation of acute respiratory distress syndrome (ARDS) after consumption of trehalose^11^; therefore, the use of trehalose is recommended to prevent COVID‐19 and to treat mild to moderate cases of the disease but not to treat patients with severe COVID‐19 and those with cytokine syndrome or ARDS. To fully ensure safety and efficacy and possible side effects, it is recommended further studies be performed on the effects of trehalose on SARS‐CoV‐2 infection.

## FUNDING INFORMATION

The authors received no financial support for the publication of this article.

## AUTHOR CONTRIBUTIONS

Writing—Original Draft Preparation: Mojde Pajokh.

Writing—Review and Editing: Mojde Pajokh, Mohammad Pourfridoni.

All authors have read and approved the final version of the manuscript.

## Data Availability

Data sharing is not applicable to this article.
